# Raoult’s law revisited: accurately predicting equilibrium relative humidity points for humidity control experiments

**DOI:** 10.1107/S1600576717003636

**Published:** 2017-03-29

**Authors:** Michael G. Bowler, David R. Bowler, Matthew W. Bowler

**Affiliations:** a Department of Physics, University of Oxford, Keble Road, Oxford OX1 3RH, UK; b Department of Physics and Astronomy, University College London, Gower Street, London WC1E 6BT, UK; c European Molecular Biology Laboratory, Grenoble Outstation, 71 avenue des Martyrs, CS 90181, Grenoble F-38042, France; d Unit for Virus Host Cell Interactions, Université Grenoble Alpes–EMBL–CNRS, 71 avenue des Martyrs, CS 90181, Grenoble F-38042, France

**Keywords:** controlled dehydration, macromolecular crystallography, Flory–Huggins entropy, statistical mechanics, humidity control

## Abstract

The humidity surrounding a sample is an important variable in scientific experiments. Biological samples in particular require not just a humid atmosphere but often a relative humidity (RH) that is in equilibrium with a stabilizing solution required to maintain the sample in the same state during measurements. The controlled dehydration of macromolecular crystals can lead to significant increases in crystal order, leading to higher diffraction quality. Devices that can accurately control the humidity surrounding crystals while monitoring diffraction have led to this technique being increasingly adopted, as the experiments become easier and more reproducible. Matching the RH to the mother liquor is the first step in allowing the stable mounting of a crystal. In previous work [Wheeler, Russi, Bowler & Bowler (2012). *Acta Cryst.* F**68**, 111–114], the equilibrium RHs were measured for a range of concentrations of the most commonly used precipitants in macromolecular crystallography and it was shown how these related to Raoult’s law for the equilibrium vapour pressure of water above a solution. However, a discrepancy between the measured values and those predicted by theory could not be explained. Here, a more precise humidity control device has been used to determine equilibrium RH points. The new results are in agreement with Raoult’s law. A simple argument in statistical mechanics is also presented, demonstrating that the equilibrium vapour pressure of a solvent is proportional to its mole fraction in an ideal solution: Raoult’s law. The same argument can be extended to the case where the solvent and solute molecules are of different sizes, as is the case with polymers. The results provide a framework for the correct maintenance of the RH surrounding a sample.

## Abstract 2

The equilibrium relative humidity values for a number of the most commonly used precipitants in biological macromolecule crystallization have been measured using a new humidity control device. A simple argument in statistical mechanics demonstrates that the equilibrium vapour pressure of a solvent is proportional to its mole fraction in an ideal solution (Raoult’s law). The same argument can be extended to the case where the solvent and solute molecules are of different sizes.

## 1. Introduction   

Sample environments that control relative humidity (RH) are important in many experiments where a wide variety of samples require specific RH values to maintain sample integrity or RH is a parameter to be varied. Humidity control has been an important parameter in the study of lipid bilayers (Lin *et al.*, 2007 ▸) and amyloid fibres (McDonald *et al.*, 2008 ▸), and in small-molecule crystallography (Mo & Ramsøskar, 2009 ▸), coherent X-ray diffraction microscopy of cells (Takayama & Nakasako, 2012 ▸) and serial crystallography (Roedig *et al.*, 2016 ▸). In biological crystallography, changing the RH can sometimes induce phase changes in crystals of macromolecules with a concomitant improvement in the quality of observed diffraction. This has been observed since the earliest days of macromolecular crystallography (Berthou *et al.*, 1972 ▸; Einstein & Low, 1962 ▸; Huxley & Kendrew, 1953 ▸; Perutz, 1946 ▸) and is most easily effected by altering the molar fraction of water in the crystal solution or by changing the RH of the air surrounding a crystal. Many successful examples are given in the literature (Adachi *et al.*, 2009 ▸; Bowler *et al.*, 2006 ▸; Cramer *et al.*, 2000 ▸; Fratini *et al.*, 1982 ▸; Gupta *et al.*, 2010 ▸; Heras *et al.*, 2003 ▸; Hu *et al.*, 2011 ▸; Kadlec *et al.*, 2011 ▸; Kuo *et al.*, 2003 ▸; Nakamura *et al.*, 2007 ▸; Sam *et al.*, 2006 ▸; Vijayalakshmi *et al.*, 2008 ▸; Yap *et al.*, 2007 ▸; Zerrad *et al.*, 2011 ▸). Several specific devices have been developed to control the humidity surrounding a crystal (Einstein, 1961 ▸; Sjögren *et al.*, 2002 ▸; Pickford *et al.*, 1993 ▸) with modern devices mounted at X-ray sources or synchrotron beamlines (Kiefersauer *et al.*, 2000 ▸; Russi *et al.*, 2011 ▸; Sanchez-Weatherby *et al.*, 2009 ▸). The ability to change the RH while characterizing changes *via* diffraction allows any changes undergone by the crystal to be observed in real time and increases the chances of characterizing a beneficial phase change.

The HC1 humidity control device was developed at the EMBL Grenoble to be a user-friendly device compatible with a complex beamline environment (Sanchez-Weatherby *et al.*, 2009 ▸). It produces an air stream with a controlled RH using a dispensing nozzle, in the same manner as cryostream devices produce a nitro­gen flow at 100 K, and is therefore easy to integrate with most diffractometers. It supplies a stream of humid air at an RH determined by a dew point controller acting on a water-saturated air supply. The device is now installed at laboratories and synchrotrons across the world (Bowler, Mueller *et al.*, 2015 ▸), resulting in many successful experiments (Hu *et al.*, 2011 ▸; Kadlec *et al.*, 2011 ▸; Malinauskaite *et al.*, 2014 ▸; Oliete *et al.*, 2013 ▸). The device can also be used for ambient-temperature data collection (Bowler, Mueller *et al.*, 2015 ▸; Russi *et al.*, 2011 ▸) where the RH must be matched to the mother liquor to prevent dehydration of the crystal. The first step in these experiments is to define the equilibrium point between the RH and the mother liquor of the sample. This is an essential step as it defines the starting point for the experiments and maintains the crystal in a stable environment when the mother liquor is removed. In order to facilitate this stage we measured the equilibrium RH points for a variety of solutions commonly used for the crystallization of proteins and nucleic acids (Wheeler *et al.*, 2012 ▸). This provided a starting point for most experiments and the results obtained were compared with Raoult’s law (Raoult, 1887 ▸) for the equilibrium vapour pressure of water above a solution [and for solutions of polymers, with a generalization (Bowler, Mueller *et al.*, 2015 ▸)]. The measurements made were consistently higher than those predicted by Raoult’s law and a satisfactory explanation for the discrepancy could not be found. Here, we have repeated the measurements using a device based on the HC1 but with higher precision in the control of RH. The new measurements are in very good agreement with Raoult’s law. Because of its importance, we present a simple explanation for Raoult’s law using statistical mechanics and also show how this treatment can be extended to polymer solutions, where Raoult’s law breaks down. These results illuminate the machinery underlying a long-observed phenomenon and allow the accurate prediction of humid atmospheres for specific sample requirements, applicable to a wide variety of fields.

## 2. Experimental procedures   

### 2.1. RH measurements   

Solutions of polyethylene glycol (PEG) were made gravimetrically at concentrations between 50 and 10%(*w*/*w*). Stock solutions of salts at 3 *M* were made and then diluted to reach the desired concentration. A round 600 µm Micromount (MiTeGen, Ithica, New York, USA) was mounted on either the BM14 or MASSIF-1 (Bowler, Nurizzo *et al.*, 2015 ▸; Nurizzo *et al.*, 2016 ▸) diffractometers with an HC-Lab device (Arinax, Moirons, France) mounted at a distance of 5 mm from the loop. The HC-Lab is based on the original HC1 developed at the EMBL, Grenoble (Sanchez-Weatherby *et al.*, 2009 ▸), but with improvements in the dew point controller, temperature measurement and calculation of RH. These developments have led to a device with superior control and stability of RH levels. In order to determine the equilibrium RH, 2 µl of solution were taken and a small drop placed on the loop with a pipette. The diameter of the drop was measured using specific image analysis software. The humidity was adjusted until the drop diameter was stable. This was repeated a few times until the drop diameter was stable upon initial placement on the loop. Each measurement was then repeated three times at ambient temperature.

## 3. Results   

### 3.1. Comparison of measured equilibria and predicted values   

**Figure 1:**
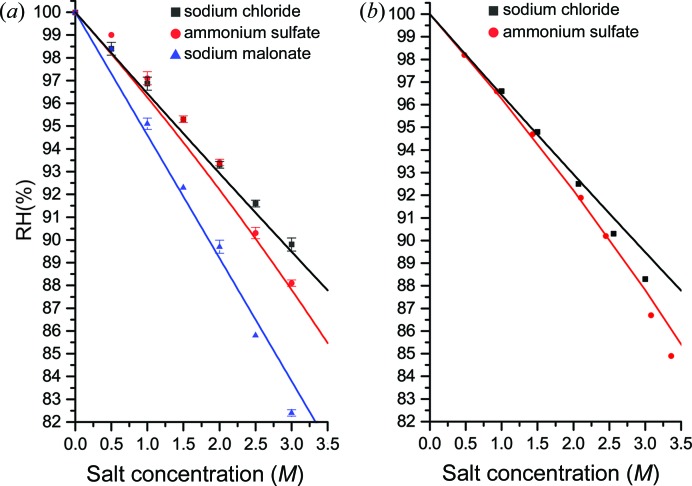
(*a*) Plots showing the equilibrium RH for salt solutions commonly used as precipitants or additives in macromolecular crystallogenesis measured using the HC-Lab. (*b*) The measured vapour pressures above solutions of ammonium sulfate (Wishaw & Stokes, 1954 ▸) and sodium chloride (Robinson, 1945 ▸). The lines show the calculated RH from Raoult’s law (Wheeler *et al.*, 2012 ▸). The measurements made using the HC-Lab [panel (*a*)] more accurately reflect the predicted values from Raoult’s law.

In previous work we measured the RH equilibrium points for a range of solutions commonly used in protein crystallization and examined the results in terms of Raoult’s law and the Flory–Huggins model for the entropy of mixing of polymers (Bowler, Mueller *et al.*, 2015 ▸; Wheeler *et al.*, 2012 ▸). While the measured values provided a starting point for humidity control experiments and Raoult’s law should be a good explanation for the observed results, there was a considerable discrepancy between the two (Wheeler *et al.*, 2012 ▸). Measured values were consistently 1–3% higher than those predicted, which was attributed to the condenser used in the device being rather inaccurate at humidity values above 96%. Repeating these measurements using the new humidity control device, the HC-Lab, the discrepancy is no longer significant (Figs. 1 ▸ *a* and 2 ▸ *a*). The results obtained from the HC-Lab are also in agreement with detailed studies of the activity of water above salt (Robinson, 1945 ▸; Wishaw & Stokes, 1954 ▸) and polymer solutions (Sadeghi & Shahebrahimi, 2011 ▸; Sadeghi & Ziamajidi, 2006 ▸) (Figs. 1 ▸ *b* and 2 ▸ *b*), with the salt solution measurements made in this study appearing to be more accurate. This now brings the control of RH surrounding crystals into line with measurements made using dedicated and accurate devices, as well as with theoretical calculations.

### 3.2. Derivation of the origin of Raoult’s law   

Raoult’s law (Raoult, 1887 ▸) describes the reduction in the saturated vapour pressure above a solvent when a mole fraction *x* of some solute is dissolved within it. If the vapour pressure above the pure solvent is *p*_0_ then the vapour pressure of the solvent above the solution is given by
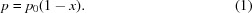
This is of course an idealization, but it is remarkably good, particularly at low mole fractions of the solute. Originally empirical, from what principles can it be derived? Any such derivations depend on the assumption of an ideal solution, meaning that within the body of the solution the elements of the solute are nearly identical to the elements of the solvent (and yet for a non-volatile solute the solute cannot enter the vapour phase). In thermodynamics, equilibrium at constant temperature and pressure corresponds to a minimum of the Gibbs’ function *G* and hence liquid–vapour equilibrium requires equal chemical potentials. The chemical potential of the solvent vapour phase is the same as that of the solvent, both above the pure liquid solvent and above a solution. The chemical potential in the solution is reduced by mixing; thermo­dynamic arguments are used to turn an entropy of mixing into a change in chemical potential. Thermodynamics does not deal with the mechanisms underlying these steps and it seems reasonable to ask, first, how the vapour pressure can be affected by the number of ways of arranging fixed numbers of two kinds of molecule and, secondly, why is there no apparent role for a work function related to the latent heat of vaporization?

Raoult’s law is the direct result of the dilution of the solvent by the solute and can be extracted by applying elementary statistical mechanics. The machinery involves the energy levels the confined components can occupy and, in the simplest case of non-ideal solutions, differences in work functions are both important and easily calculated.

#### 3.2.1. Statistical mechanics   

It is a truth universally acknowledged that any system (such as an atom in a box) that has energy levels ∊_*i*_ and is in thermal equilibrium at temperature *T* has a probability of occupying a given level proportional to 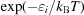, where *k*_B_ is Boltzmann’s constant, for in an ensemble the vast majority of possible configurations have this distribution and for macroscopic phenomena we are concerned with sums or averages over very many individual microscopic systems (here atoms, ions or molecules). For pure solvent we divide the energy levels into two classes, those in the liquid and those in the vapour phases. They are separated by a step in energy, a work function *W*, and so the number 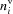, from a total of *N* atoms, found in the *i*th vapour state of energy 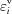 + *W* is given by
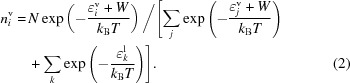
Here, the factor following the total number *N* is the probability of finding a solvent molecule in a vapour state of energy 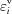 above energy *W*, and 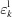 is the energy of the liquid state *k*. The sum over the index *j* in equation (2) is over the vapour states and the index *k* over the liquid states.

For a given temperature, the total number of atoms in the vapour is found by summing the numerator of equation (2) over the index *i*, yielding a fraction *y* of the total number *N*. The vapour energy levels start raised above the energy levels in the liquid by the work function *W* (closely related to the latent heat) and so the fraction of atoms in the vapour contains a suppression factor of 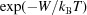. We are not yet concerned with this factor, nor with the details of the structure of the energy levels. It suffices that, for a given temperature and container, the number of atoms in the vapour phase is the fraction *y* of the total number of solvent atoms *N*. This fraction is determined by the work function, the temperature and the detailed structure of the energy levels, in turn determined by the volumes available. If a fraction *x* of the solvent atoms are removed and replaced by *Nx* units of solute, changing nothing else, the volume of the container does not change and neither the detailed structure of the energy levels nor the work function for solvent atoms changes because of the (close) identity of the solvent and solute units in an ideal solution. The fraction of solvent atoms in the vapour phase does not change and, because there are now only (1 − *x*)*N* atoms of solvent, the number of atoms of solvent in the vapour phase is reduced by a factor (1 − *x*). Hence the reduced vapour pressure and Raoult’s law.

This simple argument is indubitably correct, given the assumptions of an ideal solution. The flux of solvent molecules leaving the surface is reduced by a factor (1 − *x*), and for equilibrium both the returning flux and the number density of solvent molecules in the vapour phase are also reduced by a factor (1 − *x*), as a direct result of the lower concentration of solvent molecules. This approach can be extended to non-ideal solutions (such as solutions of polymers), but this is more complicated because of the need to calculate differences in work functions.

#### 3.2.2. Some technical details concerning volume   

A second result from elementary statistical mechanics removes a potential objection to the above argument. What if the volume of pure solvent is reduced? If the volumes of liquid and vapour are held constant, the number of vapour atoms is (for a fixed temperature) a definite fraction of the number of atoms in the liquid phase. The more general result is that the concentration of atoms in the vapour phase is a definite fraction of the concentration of atoms in the liquid phase. The vapour pressure above a liquid in a sealed container does not, in equilibrium, depend on the volume of liquid in the container. Thus (1 − *x*)*N* atoms of solvent in the container without *xN* atoms of dissolved solute would not (and does not) result in a pressure reduced by (1 − *x*). The reason is as follows. The energy levels for atoms in the vapour are those of particle waves confined within the volume between the liquid surface and the walls of the container. For an ideal gas, the number of energy levels in a given interval of energy is proportional to the volume – the spacing goes down as the volume goes up. If the volume available to vapour doubles, the number of levels in some interval Δ∊ at ∊ also doubles and hence so does the number of molecules in the vapour. Thus the concentration of atoms in the vapour phase is constant as the volume increases – the pressure remains the same. Similarly, the molecules in the liquid roam throughout the liquid volume and their wavefunctions are constrained by the walls and the liquid surface. If the volume of liquid is reduced, the sum over the populations of liquid energy levels is reduced because there are fewer of them. The spacing between energy levels in the liquid goes up with the reduction in volume and the concentration in the liquid remains the same. Thus the saturated vapour pressure above the liquid remains constant as the ratio of vapour volume to liquid volume is increased, until of course all the atoms originally in the liquid are in the vapour phase. Thereafter, as the volume is increased (by pulling back on a piston perhaps) the vapour density, and so the pressure along the isotherm, falls.

When extracted solvent molecules are replaced by solute, the solute molecules make up the missing liquid volume. This makes available to the reduced number of solvent molecules the same energy level structures in both the liquid and vapour phases. This dependence of the energy-level density on the free-range volume results in the concentration of atoms in the vapour phase being a definite fraction of the concentration in the solution. This is important for considering the vapour pressure above solutions that are not ideal, for example polymers. Finally, it is essential for understanding the thermo­dynamic treatment and entropy of mixing.

#### 3.2.3. Solutions of molecules of different sizes   

Suppose now that, instead of replacing a fraction of molecules of solvent with molecules of solute pre-empting the same volume, the solute molecules require a different volume. For the case of polymers, such as polyethyl­ene glycol (PEG), the specific volume will be larger, very substantially larger for the heavier long-chain polymers. Let there be *N*_1_ molecules of solvent of specific volume *v*_1_; similarly for the solute *N*_2_, *v*_2_. The volume occupied by the liquid solution is *N*_1_ *v*_1_ + *N*_2_ *v*_2_ and the concentration of solvent molecules is less than for pure solvent occupying the same volume. The ratio of concentrations of the solvent molecules in the solution to pure solvent gives a factor in the vapour pressure ratio of
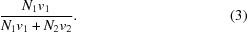
This factor reduces to Raoult’s law as the specific volumes of solvent *v*_1_ and solute *v*_2_ approach equality. This is not the whole story because the work needed to remove a solvent molecule from solution is not equal to that required to remove a solvent molecule from pure solvent, except in this limit. The following simple calculation yields the requisite difference in work functions. The work function is the work that has to be done when removing a molecule against the cohesive forces in the liquid and any contribution from ambient pressure. Because the forces are cohesive, the removal of a volume Δ*V* of liquid to the vapour state requires energy −*P*_c_Δ*V*, where the quantity *P*_c_ is the potential energy density associated with the cohesive forces. It is a contribution to the pressure in the liquid and is negative. If a volume Δ*V* is instead added, it acquires negative potential energy and the work done is *P*_c_Δ*V*, where *P*_c_ is again negative. Consider the operation of replacing a molecule of solvent by one of solute. The liquid volume increases by Δ*V* = (*v*_2_ − *v*_1_) and this volume contains a negative potential energy density. The cohesive pressure term −*P*_c_ must balance that from the thermal energy density (both are of the order of 1000 atmospheres and ambient pressures permitting the liquid state are perhaps 1 atmosphere) and so is given by
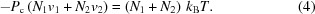
Thus the work that has to be done to make the replacement is given by
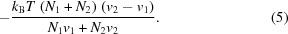
This is made up of two parts, the work necessary to insert a molecule of solute (a contribution to the chemical potential μ_2_) and the work necessary to extract a molecule of solvent (−μ_1_). The question is, what part of equation (5) is to be identified with the component of μ_1_ and, because equation (5) is a difference, what is the origin? As *N*_2_ 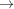 0, the solution approaches pure solvent and the term in the numerator involving *N*_2_ goes to zero, thus suggesting that the difference in the work that has to be done to deliver one molecule of solvent to the solution as opposed to pure solvent is
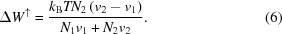
This can be verified by calculating the work done against cohesive pressure to insert a solvent molecule into the solution as opposed to the same volume of pure solvent: calculate the (pressure-related) work done inserting an atom of solvent 1 into a solution and also calculate the work done inserting an additional atom into a volume of pure species 1. In both cases Δ*v* = *v*_1_.

The magnitude of the cohesive pressure in a solution is given by
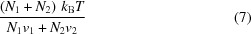
[from equation (4)] and the pressure in a pure solvent is *k*_B_ *T*/*v*_1_. Then
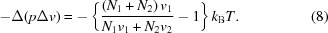
This also yields equation (6).

The difference in work functions for removing atoms to the vapour phase, 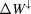, is the negative of equation (6). The effect on RH is an exponential factor
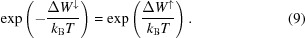
The concentration ratio of equation (3) multiplied by this factor yields the RH of the solvent:
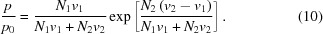
The first factor on the right-hand side is the volume fraction of solvent in the solution and reduces to Raoult’s law as the specific volumes become equal. The second factor goes to unity in this same limit. It is less obvious that equation (10) also reduces to Raoult’s law in the limit of extreme dilution, regardless of the ratio of specific volumes, but it is so.

This expression [equation (10)], derived using elementary notions from statistical mechanics, is the same as that derived using thermodynamics and the Flory–Huggins entropy of mixing devised for polymer solutions (Flory, 1942 ▸, 1970 ▸) or, equivalently, Hildebrand’s entropy of solution of molecules of different sizes (Hildebrand, 1947 ▸). In such treatments both factors in equation (10) emerge from matching chemical potentials. Our treatment clarifies the physical meaning of the factors – the first factor is the concentration ratio, while the second (exponential) factor embodies the difference in work functions arising from different specific volumes. In Appendix *A* [App appa] we discuss the relationship between simple statistical mechanics and thermodynamic arguments, addressing in particular the significance of the entropy of mixing.

#### 3.2.4. Relationship between observations and theory   

We have shown that there is good agreement between measured values of the RH above a solution and the theoretical basis for vapour pressure above a solution. How do the curves shown in Figs. 1 ▸ and 2 ▸ relate to equations (1) and (10)? The RH given by Raoult’s law is a linear function of the fraction of molecules that are solvent, the mole fraction, but the curves shown in the figures are not linear for two reasons: (i) the concentrations are shown in units that are commonly used in biochemistry, not the mole fraction, and (ii) the specific volume of the solute changes the relationship. This section explains the observed curves, first for salts and then for polymers.

Writing Raoult’s law as
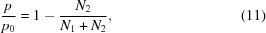
it is obvious that the RH is a linear function of the fraction of more or less freely propagating components of species 2. However, for practical reasons solutions are not usually prepared as a mole fraction, but rather of a specified molarity, the number of moles of solute in a litre of solution (the graphs in Fig. 1 ▸ are plotted as a function of molarity). If the solute molarity is to be specified, there are two complications in the translation of Raoult’s law. The first is that (ionic) salts when dissolved dissociate into freely drifting ions (such as Na^+^ and Cl^−^). The second is that the volume of water is reduced below 1 l by the volume of the salt. Thus, if *M* is the solute molarity, the RH is given by
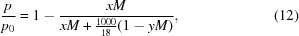
where *x* is the number of independent ions into which the salt dissociates and *y* accounts for the specific volume of the salt. For sodium chloride the quantities *x* (*y*) are 2 (0.027), for ammonium sulfate 2 (0.074) and for sodium malonate 3 (0.095). The above equation is in fact equation (3) of Wheeler *et al.* (2012 ▸). It is clear that the Raoult’s law RH is not a linear function of molarity and also that the slope at low molarity depends directly on the degree of dissociation of the salt, clearly seen in Fig. 1 ▸(*a*).

**Figure 2:**
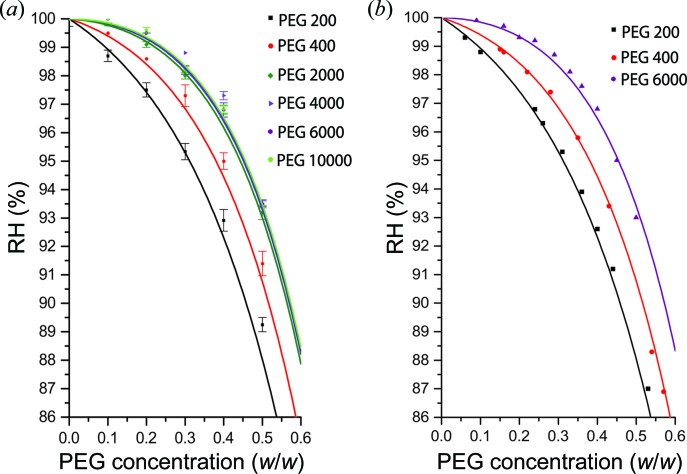
(*a*) Plots showing the equilibrium RH for PEG solutions commonly used as precipitants or additives in macromolecular crystallogenesis measured using the HC-Lab. (*b*) The measured vapour pressures above PEG solutions from Sadeghi & Shahebrahimi (2011 ▸) and Sadeghi & Ziamajidi (2006 ▸). The lines show the calculated RH from Raoult’s law modified for polymer solutions (Bowler, Mueller *et al.*, 2015 ▸).

Comparison of equations (1) and (10) makes it clear that if the specific volumes are not the same, and the solution is not very dilute, the RH is not a linear function of mole fraction. There is a further complication: equation (10) is primarily used for solutions of polymers where mass fraction (*w*/*w*), rather than mole fraction, is the most commonly used expression of concentration. Thus, to obtain the RH in the form quoted as equation (1) of Bowler, Mueller *et al.* (2015 ▸) the following steps are taken. Equation (10) is written as
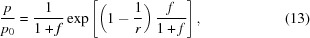
where *f* = *rN*_2_/*N*_1_ and *r* = *v*_2_/*v*_1_. The mole ratio is written in terms of the ratio of polymer mass to solution mass, here called *x* but in Fig. 2 ▸, and in other usage, weight per weight (*w*/*w*):
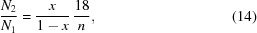
where *n* is the molecular weight of the polymer (as in PEG *n*) and 18 that of water, assumed to be the solvent.

The remaining problem is the value to be adopted for the volume ratio *r*. The expression for the RH above a polymer solution was originally worked out using the Flory–Huggins entropy of mixing. These early calculations supposed a lattice, with water molecules each occupying one site and each monomer unit of a polymer likewise occupying one site. Then, if the molecular weight of the monomer is *m* the quantity *r* is *n*/*m* and
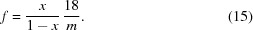
Substitution yields equation (1) of Bowler, Mueller *et al.* (2015 ▸), used in the construction of the curves in Fig. 2 ▸. (We have found that the best value of *m* for PEGs is 38.) Expressed as a function of mass fraction, the RH becomes independent of the polymer molecular mass *n* as *n* becomes very large, *i.e.* for very long chain polymers.

## 4. Discussion   

The control of the RH surrounding samples is important to maintain their integrity and study the effects of increased or decreased humidity. Here we have established that the theoretical RH values we previously calculated (Bowler, Mueller *et al.*, 2015 ▸; Wheeler *et al.*, 2012 ▸) are in satisfactory agreement with a humidity control device used on protein crystallography beamlines. As the predicted values are also in complete agreement with measurements made using specific devices, the previous discrepancies can be ascribed to shortcomings in the control of RH in the HC1c device used. We have also determined the origin of the observed vapour pressure changes above solutions of solutes. If *N* units of a liquid solvent are in an equilibrium where liquid and vapour phases coexist, a fixed fraction are (for a given temperature) in the vapour phase. If the number of units is reduced to *N*(1 − *x*) and if all else remains unchanged, because of the presence of *Nx* units of the solute in an ideal solution, then the number of units in the vapour phase (and hence the pressure) is reduced by the same factor (1 − *x*), Raoult’s law. For unequal sizes of solvent and solute components, the dilution factor has to be multiplied by an exponentiated work function. These results provide a solid basis on which to predict the RHs required to maintain a wide variety of samples and solutions in homeostasis.

## Appendix A Statistical mechanics, entropy and chemical potentials

The origin of Raoult’s law lies in the freedom of the units of solvent and solute to roam throughout the volume of liquid. For the assumptions of an ideal solution, access of both solvent and solute to the whole volume results in energy levels available (to the solvent) for a given volume, unchanged from those in the pure solvent, and the density of (energy) states is proportional to the volume. Entropy of mixing expresses these same ideas in the language of thermodynamics.

Suppose that we can decompose a system into many identical parts having energy levels ∊_*i*_. This complex system is in thermal equilibrium at some temperature *T*, with an exponential distribution in the energies of the components. Let the total energy of our complex system be *U*. The following relations apply:

and

where

A small change in the internal energy *U* can be written as

The last term vanishes if there is no change in the number of components in the system. The first term is the result of slightly redistributing the population over the energy levels ∊_*i*_. It represents the addition of heat. The second term corresponds to the energy levels changing with no change in population – doing it very slowly. If the volume slowly increases the energy levels slowly sag as the wavelengths of standing waves increase and the system does work. Thus the equivalent expression in thermodynamics is the first law in the form

where the last term is called chemical work and μ is the chemical potential. (Generally, each species of atom has its own chemical potential.)

If everything is done very slowly and reversibly,

where *S* is the thermodynamic entropy going back to Carnot. We are identifying the heat term in the first law with

Express the energy of the *i*th level in terms of its population

For fixed *N* the sum of Δ*n*_*i*_ is zero and so the term in ln(*z*) drops out and

demonstrating the equivalence of the Carnot and Boltzmann entropies.

Thus the entropy associated with *N* units (atoms, molecules, ions…) distributed over these energy levels, in equilibrium at temperature *T*, is

The probabilities *P_i_* involve the normalizing factor *z*, a sum over all energy levels. The value depends on the level density. The more levels the components are spread over, the smaller the individual *P_i_* and the larger the entropy.

Substitution of the expressions for the exponential probabilities yields for the entropy

Rewriting the sum for *z* as an integral

The density of states factor d*n*/d∊ depends on ∊ (which integrates out) and is linearly proportional to the volume available to the wandering molecules. Consider taking a volume *V*_1_ of solvent and a volume *V*_2_ of solute. (This is most easily envisaged if the solute is also a liquid; otherwise, pretend that there is a solute liquid with the properties the solute will display in the solution.) Before mixing the two together each has its entropy, appropriate to volumes *V*_1_ and *V*_2_, respectively. After mixing, both solvent and solute have access to a total volume *V*_1_ + *V*_2_. For an ideal solution nothing else has changed and so, taking the difference in entropy after and before the mixing,

This is the entropy of mixing and arises entirely from the increased density of energy levels as more volume is made available for units of both solvent and solute to roam at random. (These volumes are defined by the boundaries confining the liquids, setting boundary conditions and hence determining the quantized energy levels.)

Since we are looking at mixing of two forms of condensed matter, each with the same specific volume (an ideal solution again), the above expression for the entropy of mixing can also be written with *V*_1_ and *V*_2_ replaced by *N*_1_ and *N*_2_, respectively. The result is essentially identical to the product of *k*_B_ (Boltzmann’s constant) and the logarithm of the number of different ways of arranging *N*_1_ and *N*_2_ units (for large *N*_1_ and *N*_2_, using Stirling’s theorem). This is a purely combinatorial problem and the number of perceptibly different ways is given by

What can this have to do with the vapour pressure above a solution? We now see that *N*_1_ and *N*_2_ are (for an ideal solution) proxies for *V*_1_ and *V*_2_ and these volumes control the energy levels available to the components of the solution before and after mixing.

More generally, suppose that the solvent molecules are each associated with a free volume *v*_1_ and the solute molecules with *v*_2_. Then the entropy of mixing [equation (28) above] is

This is essentially the expression for the entropy of mixing for solvent and solute molecules of different free volumes to be found in equation (3) of Hildebrand (1947 ▸), where the volumes are introduced through a classical argument concerning uncertainty of location. It is also equivalent to the Flory–Huggins entropy for polymer solutions, most clearly discussed by Flory (1970 ▸).

The derivative of the entropy of mixing with respect to the number of solvent molecules within the solution (*N*_1_) yields the difference in chemical potentials that must match the difference in chemical potential of the vapours above the solution and the pure solvent:

For the solution, the derivative of *S*_12_ in equation (30) with respect to *N*_1_ is

and for the pure solvent before mixing the derivative of *S*_1_ is

In the standard thermodynamic argument (*e.g.* Hildebrand, 1947 ▸), taking the difference in chemical potentials and matching to the vapour phase eventually yields

which is equation (6) given by Hildebrand (1947 ▸). Then the RH of the solvent above such a solution is

Equation (35) is identical to equation (10).

In §3.2.3 we calculated the difference in work functions for the solvent in a solution of volume *V* and for the pure solvent in the same volume. This result can also be obtained from the differential of the difference in entropies of the solution and the pure solvent in equal volumes. The only terms that survive in the difference are (*N*_1_ + *N*_2_)*k*_B_ln*V* for the solution and  for the pure solvent. The volume *V* is given by

The relevant difference in chemical potentials is then

The negative of this is the difference in work functions, needed to complete the ratio of vapour pressures at the end of §3.2.3. The result given in equation (37) above of course agrees with equation (6).
